# Can CD34^+^CD38^−^ lymphoblasts, as likely leukemia stem cells, be a prognostic factor in B-cell precursor acute lymphoblastic leukemia in children?

**DOI:** 10.3389/fped.2023.1213009

**Published:** 2023-08-22

**Authors:** Weronika Stolpa, Agnieszka Mizia-Malarz, Magdalena Zapała, Bartosz Zwiernik

**Affiliations:** ^1^ Department of Oncology, Hematology, and Chemotherapy, Upper Silesia Children’s Care Health Centre, Katowice, Poland; ^2^ Department of Pediatrics, Medical University of Silesia, Upper Silesia Children’s Care Health Centre, Katowice, Poland; ^3^ Students’ Research Group, Department of Pediatrics, Medical University of Silesia, Katowice, Poland

**Keywords:** acute lymphoblastic leukemia, B-cell precursors, CD34, CD38, prognostic factor, children

## Abstract

**Background:**

CD34^+^CD38^−^ lymphoblasts as likely leukemia stem cells (LSCs) may be responsible for a worse response to treatment and may be a risk factor for recurrence in B-cell precursor acute lymphoblastic leukemia (BCP-ALL).

**Objective:**

The study objective was to assess the prognostic role of CD34^+^CD38^−^ lymphoblasts in bone marrow on the day of BCP-ALL diagnosis.

**Methods:**

115 patients with BCP-ALL, the median age of 4.5 years (range 1.5–17.9 years), gender: female 63 (54.8%) with BCP-ALL were enrolled; Group I (*n* = 90)—patients with CD34^+^CD38^+^ antigens and Group II (*n* = 20)—patients with CD34^+^CD38^−^ antigens on the lymphoblast surface.

**Results:**

A worse response on Days 8, 15, and 33 of therapy and at the end of treatment in Group II (CD34^+^CD38^−^) was more often observed but these differences were not statistically significant. A significantly higher incidence of BCP-ALL recurrence was in Group II.

**Conclusions:**

1. In BCP-ALL in children, the presence of CD34^+^CD38^−^ lymphoblasts at the diagnosis does not affect the first remission.
2. In BCP-ALL in children, the presence of CD34^+^CD38^−^ lymphoblasts at the diagnosis may be considered an unfavorable prognostic factor for disease recurrence.
3. It is necessary to further search for prognostic factors in BCP-ALL in children.

## Introduction

B-cell precursor acute lymphoblastic leukemia (BCP-ALL) is the most common neoplasm in children (1, 2). Intensive chemotherapy currently allows for overall survival (OS) in about 80% of cases at the first diagnosis. OS decreases significantly to about 50% in the case of disease recurrence (2–4).

A wide panel of diagnostic tests and the response to remission-inducing treatment allow us to distinguish risk groups in BCP-ALL. Based on the classification into risk groups, treatment is planned and the risk of treatment failure is estimated. Are there any new markers determined at the diagnosis, the presence of which would have prognostic significance?

According to some authors, the precursor cells of the leukemic clone are leukemia stem cells (LSCs) (5–13). LSCs frequently express CD34 antigen (CD34^+^) on the surface and often lack CD38 (CD38^−^) (6, 10, 14). The CD38 antigen, which is often absent on LSCs, is usually expressed on more mature B-cell precursor cells in the bone marrow. The extent of CD38 expression is proportional to the degree of maturation of B-cell precursor cells in the bone marrow (14, 15). While the “older” cells of the leukemic clone, which are hierarchically organized and after multiple divisions have programmed apoptosis, leukemia stem cells (LSCs), which have an unlimited ability to renew and are resistant to chemotherapy, can also (apart from genetic and molecular conditions) cause a poor response to treatment and recurrence of leukemia (5–7).

Could the lack of expression of the CD38 antigen on lymphoblasts in B-cell precursor acute lymphoblastic leukemia correspond to forms that are closer to LSCs, and thus less mature and more resistant to treatment?

The study objective was to evaluate the prognostic significance of the CD38 antigen on lymphoblasts in BCP-ALL in children. The response to treatment during the induction phase of remission of acute lymphoblastic leukemia (Days 8, 15, and 33 of chemotherapy) and at the end of treatment was assessed. Patients were also analyzed for disease recurrence.

## Subjects and methods

We retrospectively reviewed the medical records of 138 pediatric patients with BCP-ALL diagnosed between January 2002 and March 2021 in the Oncology, Hematology, and Chemotherapy Department of the Upper Silesia Children's Healthcare Center in Katowice, Poland.

The diagnosis was based on the French-American-British (FAB) morphological classification and cytochemical, immunophenotypic, cytogenetic, and molecular bone marrow analyses. Immunophenotyping was performed using fluoro-flow cytometry (2). The following monoclonal antibodies were included in the immunophenotyping panel: CD2, cyt CD3, CD3, CD5, CD7, CD10, CD19, CD20, CD34, CD117, CD13, CD14, CD15, CD16, CD33, CD38, CD45, CD56, CD66, MPO, and HLA-DR.

Positive expression was considered cell antigen CD34 expression ≥20% and cell antigen CD38 expression ≥30%.

The inclusion criteria were: B-cell precursor acute lymphoblastic leukemia with CD34+CD38+or CD34+CD38- lymphoblasts, and age of diagnosis ≥1 year ≤18 years old. The exclusion criteria were: T-cell acute lymphoblastic leukemia, newborns, and infants (under 1 year of age).

From all the analyzed records we included 115 patients with BCP-ALL, the median age at diagnosis was 4.5 years (range 1.5–17.9 years); there was female predominance (male/female 45.2 vs. 54.8%). Three children (2,2%) suffered from extra-central nervous system involvement at disease onset. The number of WBC on the day of diagnosis was at the average level of 26.2 G/L (range: 1.1–166.3 G/L); 43 children (31.2%) had WBC above 20 G/L. The clinical characteristics of all analyzed patients are summarized in Table 1.

**Table 1:** Comparison between the CD34^+^CD38^+^ and CD34^+^CD38^−^ groups*.*

Characteristics	General (*n* = 115)	BCP-ALL		*p*
		Group I (CD34^+^CD38^+^) (*n* = 95) median (25–75Q) (%)	Group II (CD34^+^38^−^) (*n* = 20) median (25–75Q) (%)	
Age (years) median (range)	4.5 (1.5–17.9)	4.0 (1.5–17.9)	5.5 (2.0–16.0)	NS
Sex (M/F)	52/63 (45.2%/54.8%)	43/52 (45.3/54.7%)	9/11 (45.0/54.7%)	NS
t (9;22)(+)/t (9;22)(−)	6/109 (5.2/94.8%)	5/90 (5.6/94.7%)	1/19 (5.0/95.0%)	NS
Trisomy 21(+)/Trisomy 21(−)	4/111 (3.5/96.5%)	4/91 (4.2/95.8%)	0/20 (0/100%)	NS
t (12;21) (+)/t (12;21) (−)	6/109 (5.2/94.8%)	5/90 (5.3/94.7%)	1/19 (5.0/95.0%)	NS
Risk group SRG/IRG/HRG	40/49/26 (34.8/42.6/22.6%)	37/39/19 (39.0/41.0/20.0%)	3/10/7 (15.0/50.0/35.0%)	NS
Day 8 PGR/PPR	82/33 (71.3/28.7%)	70/25 (73.7/26.3%)	12/8 (60.0/40.0%)	NS
Day 15 Bone marrow M1/M2/M3	86/19/10 (74.8/16.5/8.7%)	71/16/8 (74.7/16.8/8.4%)	15/3/2 (75.0/15.0/10.0%)	NS
Day 33 Bone marrow M1/M2 + M3	100/15 (87.0/13.0%)	85/10 (89.5/10.5%)	15/5 (75.0/25.0%)	NS
CR/non-CR First line treatment	109/6 (94.8/5.2%)	91/4 (95.8/4.2%)	18/2 (90.0/10.0%)	NS
Relapse	16/99 (13.9/86.1%)	10/85 (10.5/89.5%)	6/14 (30.0/70.0%)	*p* < 0.05
CR/non-CR at the end of the follow-up	104/11 (90.4/9.6%)	86/9 (90.5/9.5%)	18/2 (90.0/10.0%)	NS

(Ph(+), Philadelphia chromosome-positive; Ph(−), Philadelphia chromosome-negative; SRG, standard risk group; IRG, intermediate risk group; HRG, high-risk group; PGR, prednisone good response; PPR, prednisone poor response; bone marrow M1 < 5% lymphoblasts; bone marrow M2 ≥ 5 < 25% lymphoblasts; bone marrow M3 ≥ 25% lymphoblasts; CR, complete remission; non-CR, complete remission not achieved.

The whole study group (*n* = 115) was divided into 2 groups: Group I, *n* = 95 patients with CD34^+^CD38^+^; Group II, *n* = 20 patients with CD34^+^CD38^−^.

The patients received first-line chemotherapy treatment according to ALL BFM 95, ALL IC 2002, ALL IC BFM 2009, and AIEOP-BFM ALL 2017 chemotherapy protocols, depending on the year of the diagnosis and the obligatory treatment protocol in Poland. The patients were classified into risk groups (standard, intermediate, and high risk) according to the age at diagnosis, immunophenotyping, cytogenetic and molecular results, white blood cells (WBC) count at diagnosis, response to the 7-day steroid therapy, and results of the bone marrow evaluation at the 15 and 33 days of induction treatment. The maintenance treatment was based on applied chemotherapy protocols. The definition of relapse after the first complete remission was infiltration above 5% of blast cells in the bone marrow and/or blasts in extra-medullary sites. Relapse classification defined as very early relapse (VER), early relapse (ER), and late relapse (LR) was based on the time of relapse after initial diagnosis: under 18 months, between 18 and 36 months, and above 36 months respectively. The cut-off of patients’ inclusions point of follow-up was defined as March 2021.

## Statistical analysis

STATISTICA 13.0 was used for data analysis. The Student's *t*-test was used for comparing numerical data, the chi-squared test was used for comparing non-numerical dates, and *p*-values of <0.05 were considered significant.

## Results

The analysis of the study groups, taking into account age and sex, showed no significant differences. In Group I (CD34+CD38+), the fewest patients were in the HRG (19 pts; 20.0%), while in Group II (CD34+CD38-), the fewest patients were in the SRG (3 pts; 15.0%). However, the differences between the study groups depending on the risk group were not statistically significant (*p* > 0.05) (Table 1).

A retrospective analysis of selected genetic abnormalities (trisomy 21, t (9;22), t (12;21)) also showed no statistically significant differences between the groups (*p* > 0.05) (Table 1). Other genetic/molecular abnormalities were found in individual cases and were not analyzed (TCF3-HLF—2 pts, CDKN2A/B—1 pt, IKZF1—1 pt, inv 9—1 pt).

The analysis of the response to remission-inducing treatment showed that a higher percentage of patients from Group I compared to Group II had a good response to steroid therapy (PGR) (73.7 vs. 60.0%). Similarly, cytomorphological remission in the bone marrow on Day 33 of treatment was higher in Group I than in Group II (89.5 vs. 75.0%). However, the observed trend towards a better response to treatment in Group I was not statistically significant (*p* > 0.05) (Table 1).

**Figure 1:**
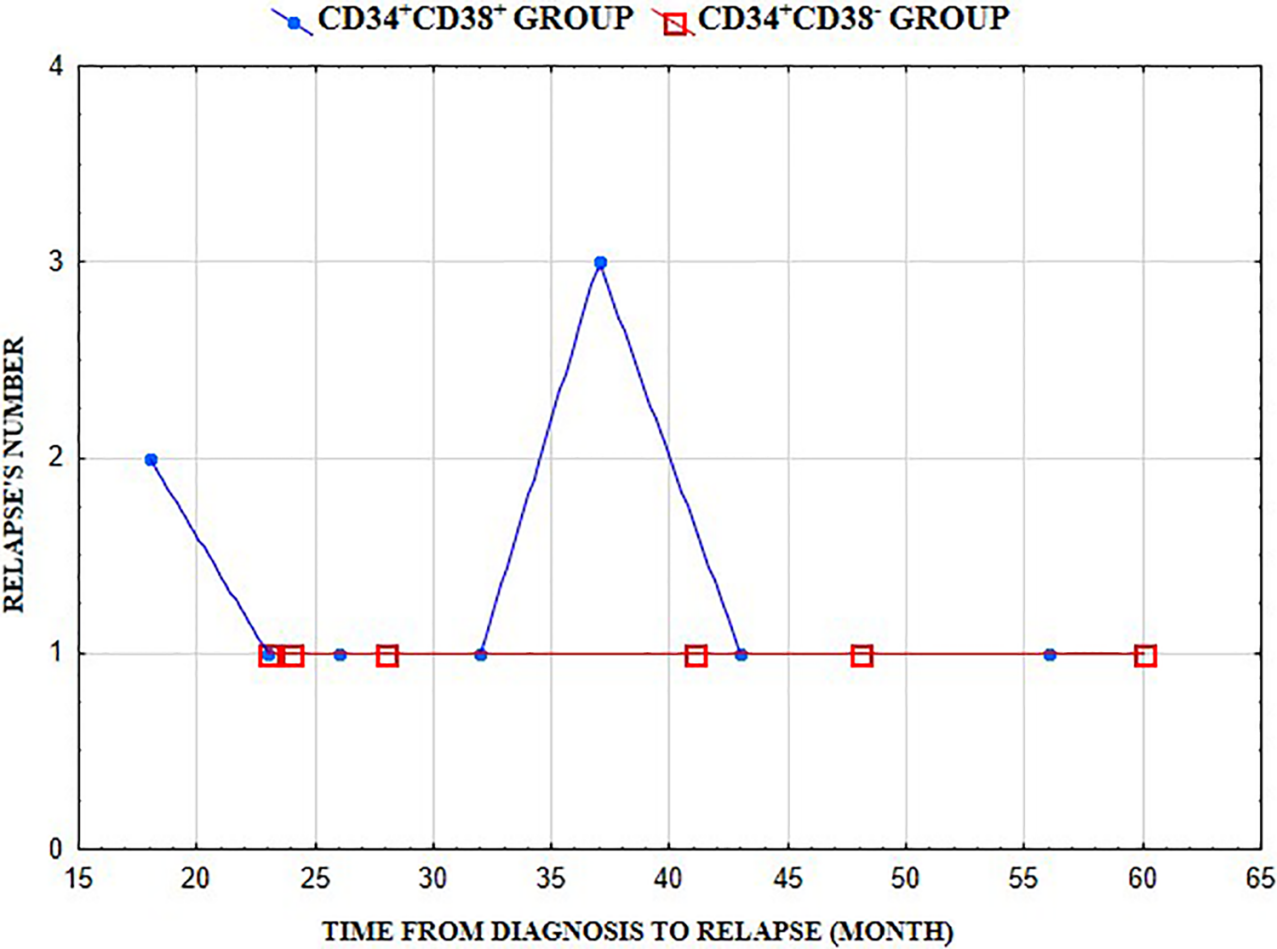
Relapse curves for the CD34+CD38+ and CD34+CD38− groups at different time from diagnosis.

The analysis of BCP-ALL recurrence showed that 10 patients in Group I (10.5%) had a recurrence of leukemia, including 5 ER and 5 LR patients. In Group II, recurrence was diagnosed in 6 patients (30.0%), including 3 ER patients and 3 LR patients. Compared to both groups, the percent of relapses was significantly higher in Group II (*p* < 0.05) (Table 1). In Group I, we observed a peak in the LR, but in Group II we did not observe a peak in types of recurrences (Figure 1). In Group II, 2/6 patients with recurrence received second-line treatment, 3/6 bone marrow transplantation (BMT), and 2/6 chimeric antigen receptor—T (CAR-T) therapy (1 pt after second BMT).

## Discussion

Relatively good treatment results in pediatric BCP-ALL do not release us from research that may have an impact on their further improvement. The presence of the CD34^+^ antigen and the absence of CD38^−^, assessed by the authors, is taken into account in the identification of leukemia stem cells. CD34^+^CD38^−^ cells are characterized by the ability to uncontrolled self-renewal and resistance to chemotherapy (3, 5–8, 18–21). The expression of the CD34^+^ antigen and the absence of CD38 observed on LSCs is also typical of normal hematopoietic stem cells (HSCs) (6, 10, 14). The distinction between LSCs and HSCs is not clear. According to Naik et al. (14), unlike HSCs, LSCs may present CD2, CD7, CD11b, CD19, CD22, and CLL-1 antigens on their surface. On the other hand, CD13, CD33, and HLA DR can be present on HSCs but not on LSCs. According to Blatt et al. (9), LSCs are characterized not only by CD34^+^, and CD38^−^ but also by CD25^+^, CD 26^+^, and Il-1RAP^+^, and these markers distinguish leukemia precursor cells from normal hematopoietic stem cells.

While the prognostic significance of the presence of CD38 on myeloblasts in acute myeloblastic leukemia (AML) in adults, and in multiple myeloma or chronic lymphocytic leukemia (CLL) is documented, the role of CD38 in BCP-ALL is unclear (9–17).

The ability of LSCs to induce the development of leukemia in severely immunodeficient mice has been proven (22). An increasing number of papers indicate the possible involvement of LSCs in the development of acute lymphoblastic leukemia (ALL) in children (23–27).

According to Ebinger et al. (24), who studied a group of 42 children with BCP-ALL and T-ALL (T-cell acute lymphoblastic leukemia), a high percentage of CD34^+^CD38^−^ on the day of diagnosis may be related to poor response to treatment on Day 33 and at week 12 of therapy in children with B-cell leukemia (24). The results of this analysis may indicate the resistance of CD34^+^CD38^−^ lymphoblasts to chemotherapy. Similarly, van Rhenen et al. (28, 29) showed that a high percentage of CD34^+^CD38^−^ correlated with high minimal residual disease (MRD) on Day 15 of therapy.

Long et al. (3) studied a group of 112 children with ALL, including 75 patients with BCP-ALL, and the correlation between cells phenotypically corresponding to LSCs and biological factors such as age, WBC (white blood cells), unfavorable genetic and molecular results, risk groups and response to treatment on Day 33. As in previous studies, the authors of this study also showed a significantly worse response to treatment on Days 8, 15, and 33 of treatment in the group of CD34^+^CD38^−^ children with BCP-ALL. In addition, a retrospective analysis showed that a high percentage of these cells at diagnosis indicated a significantly worse overall survival (OS). In our study, patients with CD34^+^CD38^−^>15% had OS of approximately 30%, while patients with CD34^+^CD38^−^<5% had OS of over 60% (3). A significant correlation between a high percentage of CD34^+^CD38^−^ cells on the day of diagnosis and the level of MRD-positive (MRD ≥ 0.01%) on Days 15 and 33 of treatment was also observed in the study by Shman et al. (31).

The results of our study did not confirm significant differences in the classification into risk groups and the response to remission-inducing treatment, or achieving remission after the end of the first-line treatment between the groups of patients with CD34^+^CD38^+^ and CD34^+^CD38^−^ lymphoblasts assessed at the diagnosis of BCP-ALL. In the group with CD34^+^CD38^−^ lymphoblasts (potentially LSCs), a higher percentage of patients qualified for HRG, a higher percentage of patients with a poor response to steroid therapy on Day 7 of treatment, no remission on Days 15 and 33 of treatment, and no remission after first-line treatment were observed. However, these differences were not statistically significant, which requires the continuation of the study on a larger group of patients.

A high percentage of cells with a phenotype that may correspond to LSCs, which appear to be more chemoresistant, results in a slower response to treatment and increases the risk of leukemia recurrence (31). According to some authors, very immature cells, such as CD34^+^CD38^−^ lymphoblasts, are less sensitive to cell cycle-specific drugs and may overexpress the multidrug transporter gene, which may result in very rapid elimination of cytostatics and thus a weaker therapeutic effect of cytostatics (23, 32, 33). Kong et al. (29) showed that recurrence in Ph(+) ALL is the result of LSCs survival after treatment. In our study, 15 patients in a group of 80 patients with Ph(+) ALL after allo-hematopoietic stem cell transplantation had CD34^+^CD38^−^ lymphoblasts in the first immunophenotypic examination. The authors showed that three months after bone marrow transplantation, an increase in the BCR/ABL transcript was observed in this group of 15 patients, which correlated with a high 3-year cumulative incidence of relapse and worse leukemia-free survival and overall survival (30). Similar conclusions are presented by Shram et al. (31).

Our analysis, similarly to the above, showed that in the CD34^+^CD38^−^ group disease recurrence was significantly more frequent. This may indicate resistance to treatment and the possibility of survival of lymphoblasts with the above immunophenotype. Taking into account the results of our study and the authors’ reports, the presence of CD34^+^CD38^−^ may be considered a predictive marker in BCP-ALL, which could be used to individualize the treatment in children with this type of neoplasm (3).

The authors of the paper would like to emphasize the fact that the above observations concern B-cell acute leukemia. A different situation applies to T-ALL, where a very strong expression of agCD38 on cancer cells is usually observed both at the first diagnosis and recurrence of the disease (11, 34, 35). The monoclonal antibody daratumumab remains a chance for these children. According to the authors, daratumumab was very effective in 14/15 cases of T-ALL recurrences (34). The above indicates that in addition to CD34^+^CD38^−^cells that may correspond to LSCs, there are many factors responsible for treatment failure in pediatric ALL, which requires constant research.

## Conclusions

1. In B-cell precursor acute lymphoblastic leukemia in children, the presence of CD34^+^CD38^−^ lymphoblasts at the diagnosis does not affect the first remission of the disease.
2. In B-cell precursor acute lymphoblastic leukemia in children, the presence of CD34^+^CD38^−^ lymphoblasts at the diagnosis may be considered an unfavorable prognostic factor for disease recurrence.
3. It is necessary to further search for prognostic factors in BCP-ALL in children, which may result in an even better understanding of the biology of the disease and greater individualization of BCP-ALL treatment in children.
